# Characterizing nanoscale spatiotemporal defects of multi-layered MoSe_2_ in hyper-temporal transient nanoscopy

**DOI:** 10.1515/nanoph-2025-0163

**Published:** 2025-06-24

**Authors:** Hwi Je Woo, Sung-Gyu Lee, Hansung Kim, Suyong Jung, Eun Seong Lee, Junghoon Jahng

**Affiliations:** Material Property Metrology Group, 65408 Korea Research Institute of Standards and Science (KRISS), Daejeon 34113, Republic of Korea; School of Electrical and Electronic Engineering, Nanyang Technological University, Singapore, 639798, Singapore

**Keywords:** transient scattering-type scanning near-field optical microscopy (s-SNOM), sideband-coupled generalized lock-in amplifier (GLIA), hyper-temporal, exciton, spatiotemporal

## Abstract

We directly characterize nanoscale spatiotemporal inhomogeneities of multi-layered molybdenum diselenide (MoSe_2_) in real space and time – the nanometre–femtosecond scale, attributing to local mechanical structures such as strain and surface/subsurface defects, which are critical in semiconductor and optoelectronic applications. This remarkable precision is achieved through the development of a hyper-temporal transient nanoscopy incorporating a sideband-coupled generalized lock-in amplification technique, allowing for characterization of local spatiotemporal defects at each pixel within a subwavelength mapping region. By utilizing this technique, we characterize the nanoscale strain-induced spatiotemporal defects of multi-layered MoSe_2_, including nano-bubbles that exhibit a noticeable reduction in exciton-exciton annihilation rates, which may attribute to the suppressed probability of bimolecular interaction of excitons due to the strain-induced band distortion. Moreover, we visualize topographically hidden spatiotemporal defects such as lattice mismatches, which induce mid-gap states that traps charge carriers and thereby slow down recombination process. We propose that this hyper-temporal approach to resolving intricate spatiotemporal inhomogeneities in van der Waals materials provides significant insights into their optoelectronic properties and opens new avenues for innovative material design and characterization.

## 1 Introduction

Multi-layered transition metal dichalcogenides (TMDs) are highly versatile materials offering superior environmental stability and are easier to produce at a larger scale, compared to their monolayer, pivotal for industrial applications [1], [2]. Their study provides a bridge between single-layer physics and bulk material science, unlocking opportunities for innovative semiconductor and optoelectronic studies. Nevertheless, studying multi-layered TMDs presents significant challenges due to the complex interplay of structural, electronic, optical, and mechanical properties across layers. For example, when layers are stacked with slight rotational misalignments, moiré patterns emerge, leading to new physical phenomena such as correlated insulating states and superconductivity [3], [4]. Moreover, the carrier dynamics such as photo-excited carrier density and population decay rates exhibit nonlinear variations influenced by the structural inhomogeneities of the layers [5].

To characterize these properties, time-resolved spectroscopic techniques employing pump-probe methods are highlighted. However, conventional far-field pump-probe methods [6], [7], [8], which are limited by diffraction, restrict spatial resolution to the micrometer scale. The lack of spatial precision makes it difficult to resolve spatiotemporal variations in carrier dynamics, which are often governed by nanoscale structural inhomogeneities, such as structural defects and grain boundaries. In this point of view, the scattering-type scanning near-field optical microscopy (s-SNOM) with pump-probe technique has emerged as a powerful tool for the spatiotemporal studies, by confining the interaction volume to the nanometer scale around the sharp metallic tip apex (∼20 nm) of the s-SNOM probe [9], [10], [11], [12], [13], [14], [15], [16], [17]. This approach enables unprecedented spatial mapping of transient phenomena, providing direct visualization of ultrafast processes with respect to the nanoscale structural inhomogeneities. For instance, the characterization of exciton formation, the Mott transition under high carrier density, and strain-induced exciton-exciton annihilation (EEA) suppression has been a subject of significant interest [11], [15]. These studies have provided valuable insights into the transition from bound excitonic states to unbound electron-hole plasma, related to the local structural variation.

Despite these advancements, current pump-probe nanoscopy approaches still face significant challenges. A key challenge lies in achieving simultaneous high spatial and temporal precision across the entire dataset. Given the localized nature of nanoscale spatiotemporal defects, comprehensive measurements across entire regions are essential for a thorough understanding of multi-layered TMD systems. However, most studies to date focus on high-spatial-precision images at fixed time delays (snapshot imaging) [11], [18] or measure high-temporal-precision dynamics at individual points (point spectroscopy) [19], hampering a comprehensive spatiotemporal analysis of ultrafast processes. Consequently, these techniques are limited to study the complex systems such as multi-layered TMD devices, which often exhibit non-uniform or heterogeneous behavior. Without a comprehensive spatiotemporal dataset, systematic statistical analysis, such as the distribution of carrier lifetimes or variations in exciton dynamics by considering local structural inhomogeneities across many different regions, remains challenging and ambiguous.

Here we employ a hyper-temporal approach to s-SNOM with the sideband-coupled generalized lock-in amplifier (GLIA) technique that allows for the pixel-by-pixel acquisition of time-resolved near-field amplitude and phase in a raster scan manner. Such a method would enable fully visualizing carrier dynamics across the entire region of the complex system, providing a full spatiotemporal map of ultrafast processes at nanoscale. Our approach achieves both high spatial and temporal precisions by eliminating inefficiencies of conventional homodyne detection scheme in pump-probe nanoscopy, such as the requirement for extensive reference mirror scanning [11], [13]. This approach facilitates faster, more stable, and highly precise data acquisition, revealing patterns and correlations that are otherwise obscured in spatially or temporally limited datasets.

Theoretically, our method reduces the total acquisition time by roughly a factor of two compared to conventional two-phase homodyne detection. In practice, however, it also removes the need to locate the 0° and 90° phase positions and to compile separate time-resolved traces for each, so the real-world efficiency gain often exceeds factor of 2. Utilizing this method, we thoroughly investigate the nanoscale spatiotemporal defects in complex multi-layered TMDs, caused by factors such as lattice mismatch, mechanical strain, and nano-bubbles. We believe that this comprehensive understanding is crucial for optimizing the performance of multi-layered TMDs in advanced semiconductor and optoelectronic applications.

## 2 Results

### 2.1 Sideband-coupled GLIA method for pump-probe nanoscopy

**Figure 1:**
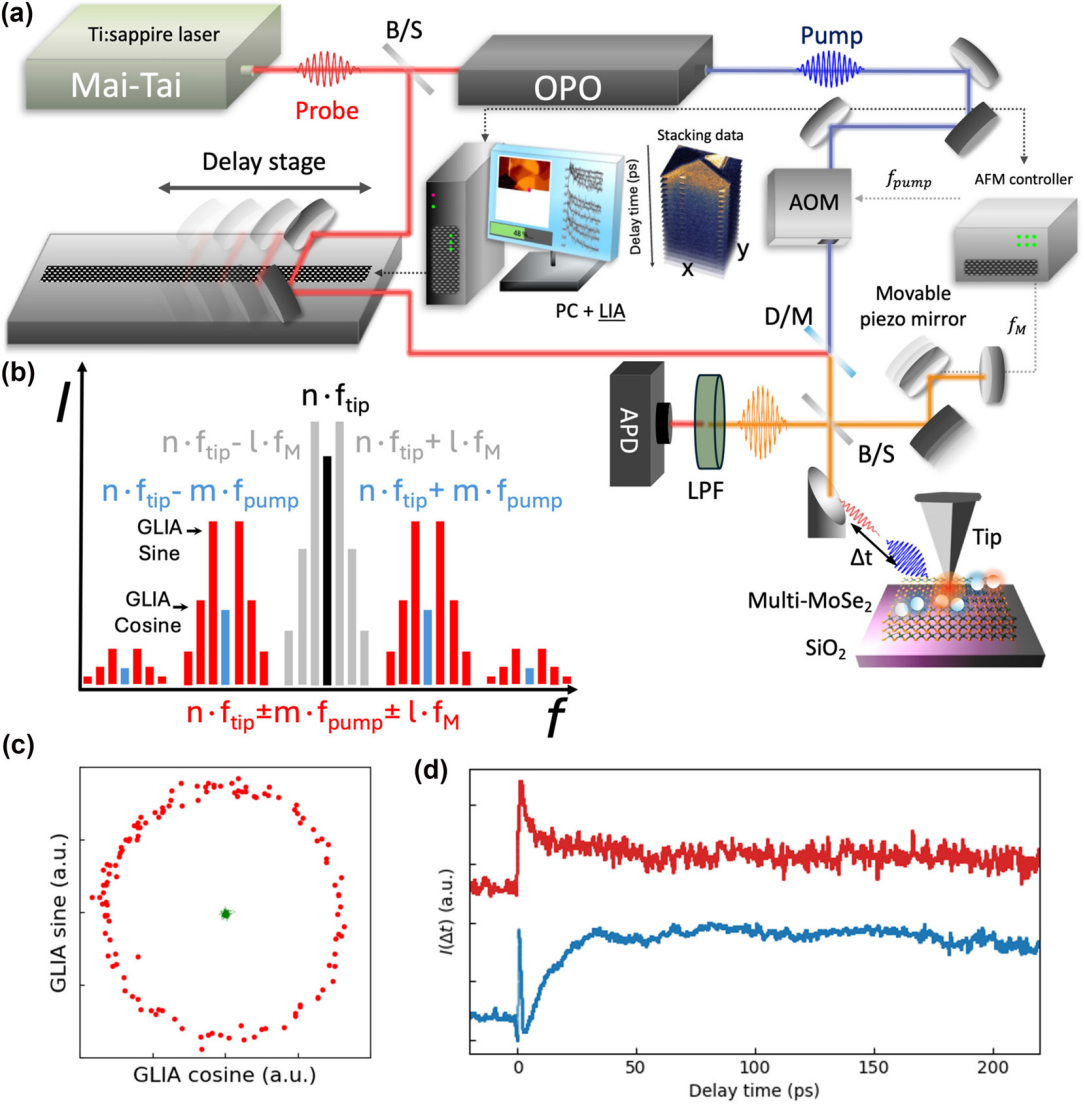
Experimental setup for sideband-coupled GLIA s-SNOM. (a) Schematic illustration of the pump-probe s-SNOM system (LIA: lock-in amplifier, D/M: dichroic mirror, B/S: beam splitter, APD: avalanche photodiode, LPF: long pass filter). (b) Frequency spectrum of s-SNOM signals, where the grey bars represent the ground state near-field signals. (c) Sideband-coupled GLIA phasors diagrams for various conditions (red: pump on and demodulated at *f*_M_ with GLIA, and green: pump off and demodulated at *f*_M_ with GLIA). (d) Time-resolved spectra measured by sideband-coupled GLIA (red) and demodulated at *f*_pump_ (blue).

The integration of the sideband-coupled GLIA technique with s-SNOM greatly enhances signal detection in pump-probe experiments. By accounting for all harmonic components of time-resolved near-field signals, it improves signal-to-noise ratio for the weak signal while effectively suppressing far-field interference [20]. Additionally, this approach eliminates the need for double scanning, a common limitation of conventional pump-probe s-SNOM setups employing homodyne detection schemes [11], [13]. In Figure 1a, the experimental setup is sketched. The system combines two tunable femtosecond pulsed lasers covering the visible-near-infrared spectral ranges. The visible pump pulse is provided by the optical parametric oscillator (OPO) output, whose intensity is modulated by the acousto-optic modulator (AOM). The near-infrared (NIR) probe pulse is provided by the Ti:Sapphire laser with no intensity modulation (DC). By adjusting the mirror on the delay stage, we control the delay time between pump and probe pulses, synchronizing with the AFM controller. The pump and probe beams are co-aligned and focused by a parabolic mirror (NA ∼ 0.4) to the tip-sample junction. The beams are reflected by a beam splitter to the reference arm, where the beam path length is modulated by a piezo-driven reference mirror vibrating at frequency *f*_M_ (typically a few hundred Hz). The back-reflected reference signal is then combined with the scattered signal from the tip-sample junction to make an interference and directed to an avalanche photodiode (APD) via a long pass filter (LPF), which blocks the pump beam to monitor the probe signal. When the pump beam (modulated at *f*_pump_) and the probe beam (DC) illuminate to the tip which oscillates at frequency of *f*_tip_ near the sample surface, the total intensity, *I* ^total^, obtained from the APD by considering the interference due to back-reflected reference signal (*E* ^*R*^) can be simplified as:

(1) $$I^{\text{total}}={\left|E^{N}+E^{F}+E^{R}\right|}^{2}$$

where the *E* ^*i*^ are the scattered near-field (*i* = *N*) and far-field (*i* = *F*) from the tip-sample junction, respectively.** **The theoretical details of the extraction of time-resolved near-field signals in pump-probe s-SNOM with sideband-coupled GLIA technique is presented in Supporting Note 1. The scattered fields can be regarded as two kinds of fields with respect to the pump-probe time delay (Δ*t*): one is the time-independent (ground-state) field from the pump ( $E_{\text{pump}}^{i}$ ) and probe ( $E_{\text{probe}}^{i}$ ) and the other one is time-resolved (transient) field ( $E_{\text{tr}}^{i}$ ) with *i* = *N* or *F*, respectively. In this regard, the total scattered fields can be considered as $E^{i}=E_{\text{pump}}^{i}+E_{\text{probe}}^{i}+E_{\text{tr}}^{i}\left(\Delta t\right)$ with *i* = *N* or *F*, respectively. Then, the time-resolved s-SNOM intensity, $I\left(\Delta t\right)$ , is determined by the time-resolved near-field $E_{\text{tr}}^{N}\left(\Delta t\right)$ , which is modulated at the sideband frequency *n* ⋅ *f*_tip_ ± *m* ⋅ *f*_pump_ [13]. This near-field is further multiplied by the reference field *E* ^*R*^ at *f*_M_, generating additional phase modulation at *n* ⋅ *f*_tip_ ± *m* ⋅ *f*_pump_ ± *l* ⋅ *f*_M_ [21], [22], [23]. Then, the amplitude $I\left(\Delta t\right)$ and phase $\phi \left(\Delta t\right)$ of the time-resolved s-SNOM signal are demodulated using the GLIA technique which simultaneously integrates the sidebands of *n* ⋅ *f*_tip_ ± *m* ⋅ *f*_pump_ signals at *f*_M_ by multiplying the two orthogonal harmonic references, respectively [20].

The frequency spectrum of the detector signal is illustrated in Figure 1b where the black bar represents the *n*th harmonic signal from tip-modulated near-field with *m* = 0 while the blue bars correspond to sideband near-field signal with *m* ≠ 0 and *l* = 0. The red bars result from interference between $E_{\text{tr}}^{N}$ and *E* ^*R*^, which are used to retrieve the $I\left(\Delta t\right)$ and $\phi \left(\Delta t\right)$ of the time-resolved near field s-SNOM signal. To validate sideband-coupled GLIA technique in the present study, the detector signal is simultaneously demodulated with GLIA while sweeping the piezo-driven reference mirror to generate a phasor diagram, $X_{I\left(\Delta t\right)}=I\left(\Delta t\right)\cos\left(\phi \left(\Delta t\right)\right)$ and $Y_{I\left(\Delta t\right)}=I\left(\Delta t\right)\sin\left(\phi \left(\Delta t\right)\right)$ . Figure 1c displays the two different cases: (1) with the pump pulse off (green dots) showing no response on the phasor diagram; and (2) with the pump pulse on (red dots) resulting in a clear circular phasor diagram, which confirms that the sideband-coupled GLIA signal is properly demodulated. To verify that the sideband-coupled method suppresses the background fields, Figure 1d shows time-resolved spectra at the same location for both sideband-coupled GLIA signal (red) and the signal demodulated at *f*_pump_ (blue). The decay curve demodulated at *f*_pump_ (blue) exhibits outstanding dips, which originate from interference between $E_{\text{tr}}^{N}$ and background fields ( $E_{\text{tr}}^{F}$ and $E_{\text{probe}}^{N}$ ). On the other hand, in the sideband-coupled GLIA conditions, the decay curve clearly shows exponential decay, indicating successful separation of the time-resolved near-field from the background fields (More details of GLIA in Supporting Notes 1 and 2).

### 2.2 Hyper-temporal s-SNOM

**Figure 2:**
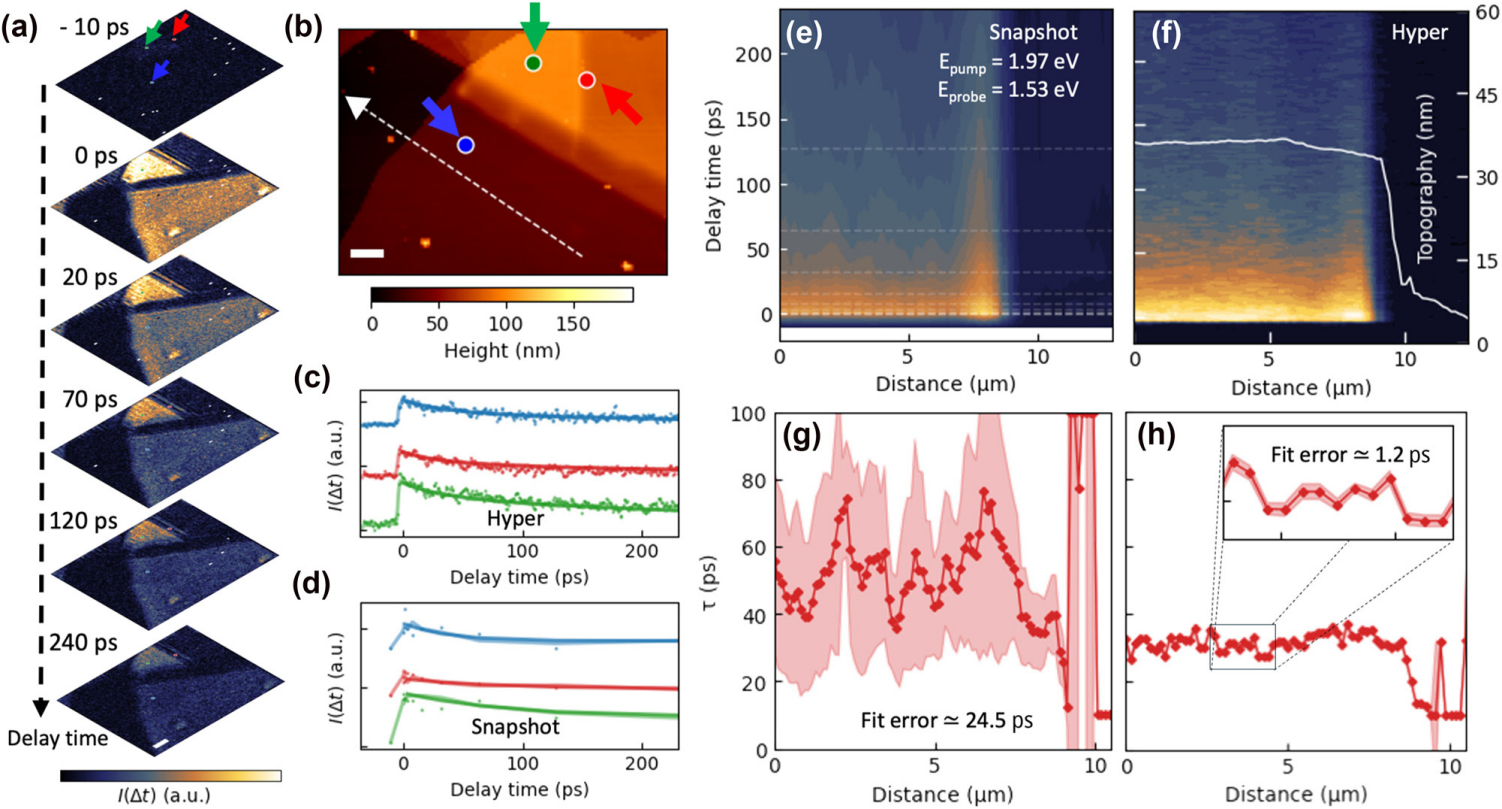
Comparison between snapshot and hyper-temporal method. (a) Time-resolved near-field images extracted from the hyper-temporal data (Extended Data 1) for specific delay times (−10, 0, 20, 70, 120, and 240 ps, scalebar: 1.5 μm). (b) AFM topographic image of multi-layered MoSe_2_ (scalebar: 1.5 μm). (c) Time-resolved spectra extracted from the hyper-temporal data (Extended Data 1). (d) Time-resolved spectra extracted from the snapshot data at colored positions. Spectra in (c) and (d) are vertically shifted for clarity. (e) The time-resolved line profile along the white dashed line in (b), extracted from the snapshot data. (f) The time-resolved line profile from hyper-temporal data. (g) and (h) Results of fitting each time-resolved line profile fitting with global fitting function *Ae* ^−Δ*t*/*τ*^, where *A* is maximum amplitude of the s-SNOM signals.

To comprehensively analyze the nanoscale spatiotemporal defects, it is essential to visualize carrier dynamics at nanoscale for every pixel within the image. In this study, we combined the hyper-temporal capabilities with the sideband-coupled transient s-SNOM system to investigate carrier dynamics in multi-layered molybdenum diselenide (MoSe_2_). High temporal resolution images were captured from a hyper-temporal dataset (Figure 2a; see Figure S2b and Extended Data 1). A subset of images from Extended Data 1, shown in Figure 2a, reveals the evolution of near-field signals at each pixel over time. The corresponding topographical map of the sample is presented in Figure 2b. The time-resolved decay curves (Figure 2c) were extracted from three distinct positions marked by the colored arrows in Figure 2b. For comparison, the transient s-SNOM images at specific time delays (snapshot imaging, Figure S2a) were also obtained in Figure 2d. The hyper-temporal approach provides significantly higher temporal precision (Figure 2c, 344 pixels) than the snapshot method (Figure 2d, 12 pixels). Although the total measurement time for the snapshot and hyper-temporal method are similar (7–8 h), the hyper-temporal method enables a more detailed analysis of how ultrafast carrier dynamics are influenced by sample inhomogeneities.

For instance, we extracted line profiles along the white dashed line in Figure 2a. As demonstrated in Figure 2e and f, the time-resolved profiles obtained with the hyper-temporal method exhibit greater clarity compared to those captured by the snapshot imaging. Exponential fitting of each pixel’s line profile (Figure 2g and h) reveals that the measured relaxation times (decay times) differ depending on the measurement methods: the hyper-temporal method yields a decay time of approximately 30 ps, while the snapshot method estimates it at around 50 ps. This discrepancy is attributed to be (1) limited time precision and (2) low spatiotemporal correlation, which are induced by a thermal drift over the long acquisition time during each** **snapshot imaging. Furthermore, the fitting accuracy is significantly improved with the hyper-temporal method, as evidenced by the fitting prediction error: the snapshot method produces a fitting prediction error of approximately 24.5 ps, whereas the hyper-temporal method reduces this error to about 1.2 ps, representing an accuracy enhancement of approximately 95 %. These clearly demonstrates that the hyper-temporal approach is essential for accurately capturing the spatiotemporal dynamics.

### 2.3 Carrier dynamics in multi-layered MoSe_2_

**Figure 3:**
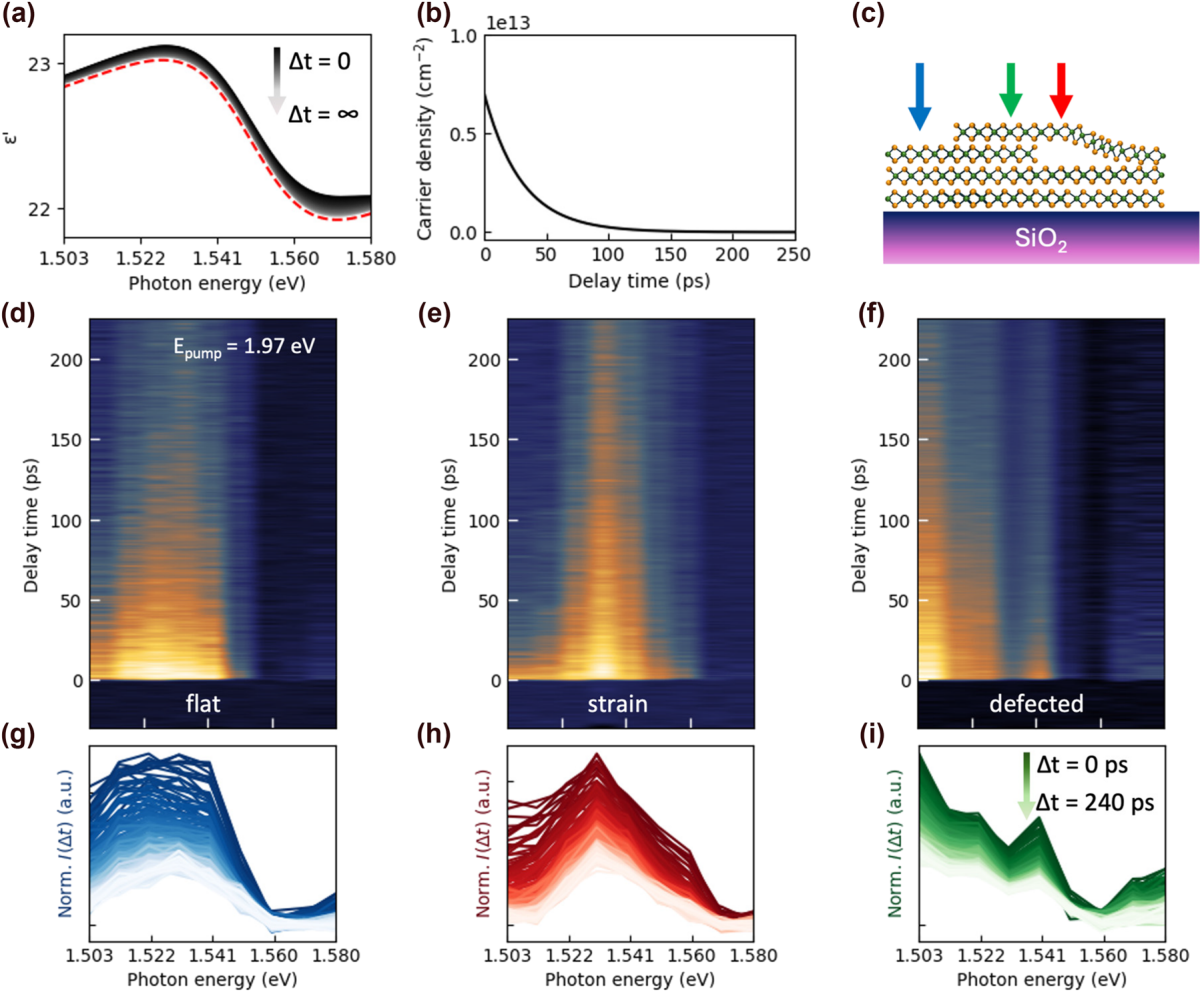
Time-resolved spectroscopy with s-SNOM for three distinct points. (a) Calculated time-resolved dielectric function of MoSe_2_ with Eq. (2) ****(red dashed line: dielectric function at infinite time). (b) Simulated carrier densities as a function of delay time. (c) The illustration of topography of each colored spots. (d)–(f) Measured time-resolved spectrum colormaps at three distinct points. (g)–(i) Corresponding normalized s-SNOM intensity for various delay time.

The s-SNOM has demonstrated to be a highly effective tool for probing excitonic properties of TMDs, particularly by accounting for their Lorentzian contributions to the dielectric function. By resolving the dielectric function at the nanoscale, it becomes possible to investigate excitonic phenomena with high spatial resolution, revealing detailed insights into their dynamics. Figure 3a illustrates analytic calculation of dielectric function changes in multi-layered MoSe_2_ at various delay times, assuming a monoexponential decay in carrier density. The temporal changes in the dielectric function are modeled using the Drude–Lorentz approach [15], [24], [25]:

(2) $$\begin{array}{ll} \varepsilon \left(E,\Delta t\right) & =\varepsilon_{\infty }^{*}+\frac{n_{\text{ex}}\left(\Delta t\right)e^{2}}{d\varepsilon_{0}\mu }\times \frac{f_{\text{osc}}}{E_{\text{ex}}^{2}-E^{2}-iE\gamma }\\ & \;-\frac{n_{fc}\left(\Delta t\right)e^{2}}{d\varepsilon_{0}\mu }\times \frac{1}{E^{2}+iE\Gamma } \end{array}$$

where *n*_ex_ is the density of photo-excited excitons, *f*_osc_ their oscillator strength, *E*_ex_ the exciton transition energy, *γ* the spectral bandwidth, *n*_fc_ the density of photo-excited free carrier density, Γ the scattering rate of free carriers, *μ* the effective reduced mass of carriers, *ɛ*_0_ permittivity in vacuum, $\varepsilon_{\infty }^{*}$ the dielectric function at infinite time, *d* the MoSe_2_ thickness, and *e* the elementary charge of electron. This model distinguishes the Lorentzian component arising from photo-excited excitons and the Drude component from free carriers, both of which modulate the complex dielectric function of the system [25]. The pump pulse excites both bound excitons and unbound free carriers in multilayer MoSe_2_, and their combined response alters the optical properties. In the visible to near-infrared spectral range for MoSe_2_, however, the Lorentzian component mostly dominates in Eq. (2). Thereby, free electrons of Drude–Lorentz model in visible-NIR range shows small number of changes of dielectric function (<0.25 %, Figure S7). In Figure 3a, the observed modulation in the calculated dielectric function (real part) is primarily due to the temporal decrease in the *n*_ex_ as shown in Figure 3b presenting the simulated carrier density. The time dependent decreases of carrier density results in a reduction of s-SNOM intensity. To connect these changes in dielectric function with s-SNOM intensity, we employed a finite dipole model suitable for multi-layered samples [26], [27], [28]. The s-SNOM intensity calculated using the finite dipole model can be expressed as:

(3) $$I\left(\Delta t\right)\propto {\left(1+r_{p}\left(\Delta t\right)\right)}^{2}\alpha_{\text{eff}}\left(\Delta t\right)E_{0}$$

The time dependent reflection coefficient $r_{p}\left(\Delta t\right)$ and effective polarizability $\alpha_{\text{eff}}\left(\Delta t\right)$ of the sample lead to changes in the intensity of time dependent near-field signals (for more details, see Supporting Note 3).

By employing transient s-SNOM with sideband-coupled GLIA, it is possible to characterize both surface and subsurface local structural inhomogeneities. As illustrated in Figure 3c–i illustrate the time-resolved carrier dynamics as a function of probe energy at three distinct locations, indicated by the colored dots in Figure 2b, which reflect mechanical defects. In the flat region (blue dot in Figure 2b and blue arrow in Figure 3c), the time-resolved spectra with respect to probe energy closely correspond to the theoretical Lorentzian model (Eq. (2)), as shown in Figure 3d and g. On the other hands, the strained region (red dot in Figure 2b and red arrow in Figure 3c) in Figure 3e and h, which resemble the Lorentzian model but show modifications due to band distortions induced by lattice strain [29]. Even in regions without evident surface distortions (green dot in Figure 2b), non-Lorentzian model behavior is observed, as demonstrated in Figure 3f and i, suggesting the presence of hidden subsurface defects such as lattice mismatches.

**Figure 4:**
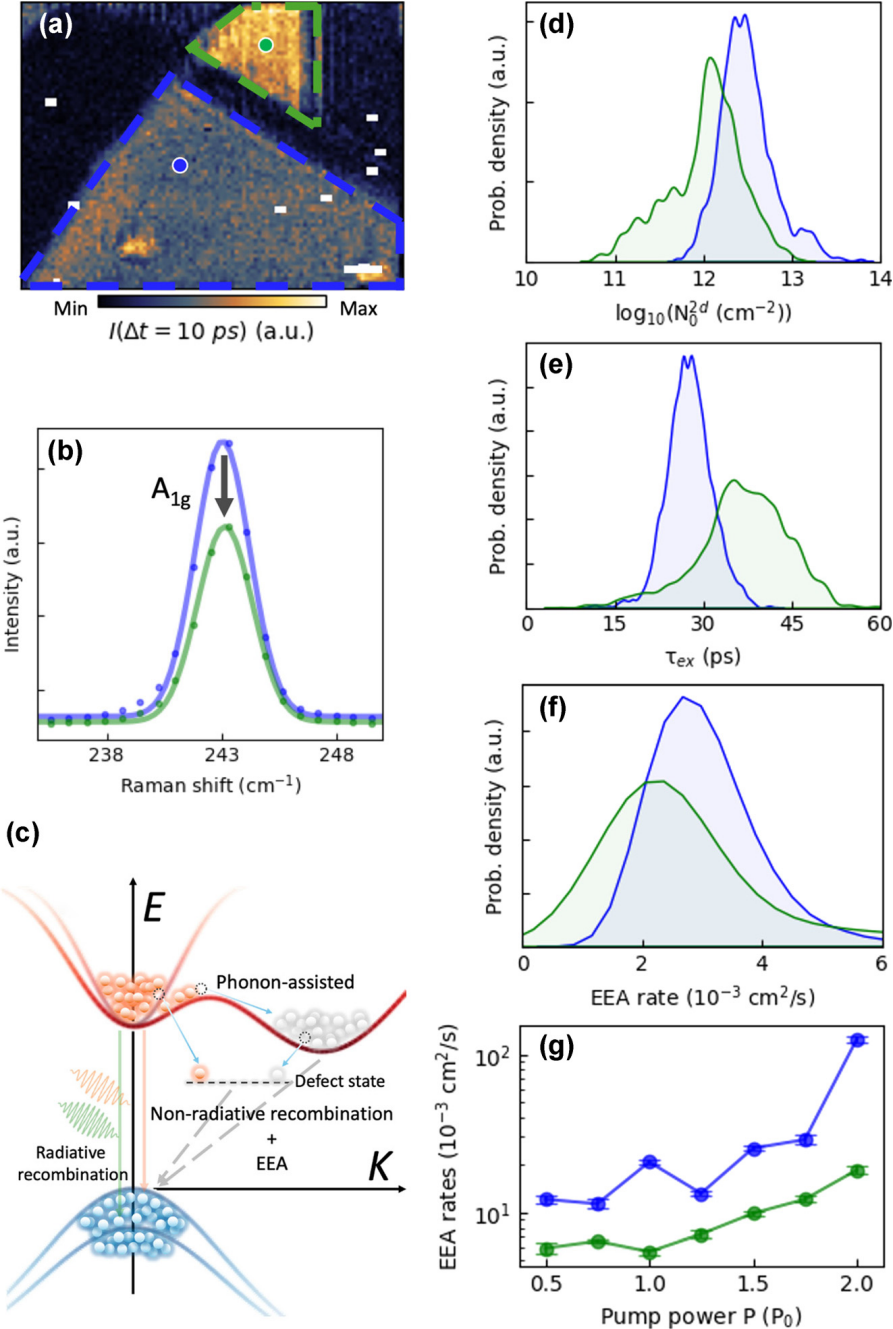
Exciton dynamics for defective and pristine regions in multi-layered MoSe_2_. (a) s-SNOM intensity at delay time 10 ps, extracted from the hyper-temporal data (scalebar: 1.5 μm). The defective region is marked by the green dashed line, and the pristine region is marked by the blue dashed line. (b) Raman spectrum from the two regions. (c) Conceptual illustration of direct and indirect bandgap, along with recombination process, including non-radiative processes. (d)–(f) Calculated probability distribution of photo-excited exciton densities, recombination time, and EEA rates based on kernel density estimation. (g) Estimated EEA rates as a function of normalized pump powers.

Unlike strain-induced inhomogeneities, such defect states can be distinguished through transient spectral analysis, as these defects can introduce mid-gap states [30], [31] that alter the electronic band structure, consistent with the observed distortions. In our measurements, shown in Figure 3i, the increase in low-energy regions of the spectrum clearly supports the presence of mid-gap states. This mechanical inhomogeneities can be further corroborated by Raman spectroscopy, comparing the flat and defect regions (blue and green dashed areas in Figure 4a). The corresponding spectra (blue and green solid lines) in Figure 4b reveal the reduced A_1g_ mode intensity in the green region, indicating the localized variations in crystal quality and defect density [32], [33], [34].

The energy band diagram (Figure 4c) conceptualizes various carrier recombination pathways, including both radiative and non-radiative processes. In multi-layered TMDs, interlayer interactions often shift the conduction band minimum away from the *K*-point while the valence band maximum remains at *K*, resulting in an indirect bandgap. This momentum mismatch reduces radiative recombination efficiency, making non-radiative pathways dominant. As shown in Figure S5a, the absence of photoluminescence indicates that non-radiative decay processes dominate in our multi-layered MoSe_2_. Such non-radiative decay processes, including phonon- or defect-assisted recombination, align well with conceptual diagram in Figure 4c.

To comprehensively understand the non-radiative exciton dynamics, we fit all hyper-temporal pixels by Eqs. (2) and (3) ****in conjunction with the rate equation (Supporting Note 4) to extract recombination times (*τ*_ex_), initial photo-induced exciton densities ( $N_{0}^{2d}$ ) and EEA rates in Figure 4d–f. Figure 4d presents the probability density function based on kernel density estimation of the initial photo-induced exciton density for the two differently colored regions. The pristine (blue) area exhibits an order of magnitude higher density (approximately 10^12^–10^14^ cm^−2^) than the defective (green) area (roughly 10^11^–10^13^ cm^−2^). The reduced $N_{0}^{2d}$ in the defective region results from structural imperfections such as atomic vacancy, or lattice mismatches. These defects distort the local band structure and induce the mid-gap states, as illustrated in Figure 4c, which trap electron-hole pairs with trapping time of approximately 1 ps [35]. This trapping state in the defective region leads to decrease the initial exciton density compared to the pristine region.

Defects have a significant impact on the exciton recombination dynamics, as shown in Figure 4e and f. The probability density function of *τ*_ex_ indicates that the defective region exhibits a longer recombination time (37.2 ps), with the *τ*_ex_ approximately 35.8 % slower than the pristine region (27.4 ps). To further clarify the influence of defects on exciton dynamics, we investigated EEA – a bimolecular process in which two excitons interact, leading to the annihilation of one exciton and the redistribution of its energy. These effects are especially pronounced at high exciton densities in the early time window (0–25 ps) [15], [36], [37]. By focusing on this interval and extracting the average EEA rates (see Supporting Note 4, Figure S6), we gained insight into how defects modulate exciton recombination under elevated carrier densities. In the pristine region, the EEA rate is estimated as about 2.90 × 10^−3^ cm^2^/s, whereas in the defective region it decreases to about 2.23 × 10^−3^ cm^2^/s. This decrease reflects a fundamental band structure that change in exciton–exciton interaction efficiency in the presence of defects. For example, spatial localization of excitons around trapping sites may reduce their interaction cross-section, or defects may introduce nonradiative pathways that compete with EEA. These effects may suppress annihilation events, resulting in a lower EEA rate constant and longer exciton lifetime in the defective regions [38], [39]. Consequently, excitons in defective regions persist longer, further increasing their lifetimes.

The promotion of EEA under higher initial carrier density conditions is demonstrated in Figure 4g. Increasing the pump fluence from 0.5 *P*_0_ to 2.0 *P*_0_ (where *P*_0_ is the reference pump fluence for the hyper-temporal mapping, *P*_0_ ∼ 0.6 mJ/cm^2^) raises the transient s-SNOM intensity, indicating an increase in photo-excited (initial) carrier density. In multilayer MoSe_2_, interlayer coupling produces much stronger dielectric screening than in the monolayer, which reduces the exciton binding energy from several hundreds of meV down to approximately several tens of meV [40]. As a result, under our experimental fluence (*P*_0_ ∼ 0.6 mJ/cm^2^), the initial carrier density approaches the Mott threshold for exciton ionization, where bound excitons begin to dissociate into a free electron–hole plasma. This transition from bound excitons to an unbound electron–hole plasma alters the recombination dynamics, enhancing nonradiative decay channels such as free-carrier Auger type recombination (*e.g.* EEA). The effective recombination coefficient increases nonlinearly near the Mott transition, giving rise to the pronounced EEA effect observed in Figure 4g [11], [15].

**Figure 5:**
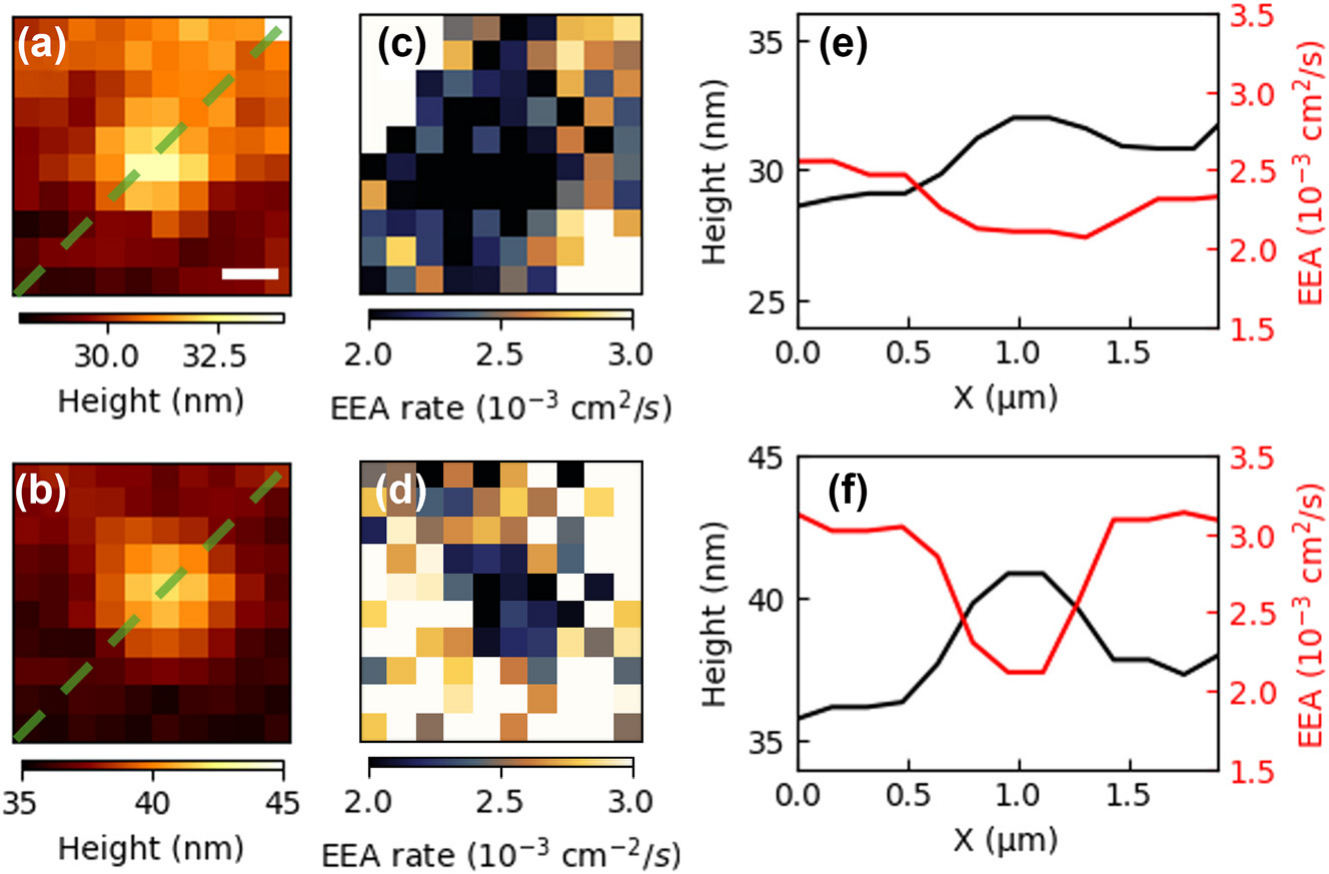
Exciton dynamics in nano-bubbles. (a) and (b) Topographic images of the nano-bubble (scalebar: 300 nm). (c) and (d) Corresponding EEA rate maps of nano-bubbles. (e) and (f) Height profiles along the green dashed lines in (a) and (b) (black) and corresponding EEA rate profiles (red).

To further explore the influence of local structural features on exciton dynamics, we focus on regions containing strain-induced nano-bubbles. Figure 5a and b display topography maps, revealing nano-bubble formations as protrusions above the flat surface. Figure 5c and d show corresponding EEA rate maps derived from hyper-temporal data. Notably, these maps visualize spatiotemporal defects with a resolution below the diffraction limit, clearly highlighting a noticeable reduction in EEA rates within the nano-bubble regions compared to adjacent flat areas. Line profiles extracted across the nano-bubble (Figure 5e and f) quantitatively demonstrate that EEA rates in these regions are reduced by approximately 20–35 % compared to the flat regions.

This suppression of EEA rates can be attributed to strain-induced modifications in the local band structure as shown in Figure 3h, which specifically affect the exciton transition energy (*E*_ex_) and its relationship to van Hove singularities in the density of states (DOS) [41]. In the strain-free regions, EEA involves two excitons that interact to produce a high-energy electron-hole pairs with total energy of 2 *E*_ex_ [41], which is strongly influenced by the DOS. When 2 *E*_ex_ aligns closely with a van Hove singularity, where the DOS increases dramatically, EEA occurs efficiently. Conversely, in the strained regions, the local band structure is distorted, shifting *E*_ex_ and altering both exciton binding and transition energies [29]. This shift displaces the final states of EEA from the van Hove singularity, reducing the DOS at the required energy level and suppressing EEA efficiency. With fewer final states available for the EEA process, the overall EEA rates decrease, consistent with the observed reduction in nano-bubble regions.

## 3 Discussion

By utilizing the hyper-temporal s-SNOM technique with sideband-coupled GLIA method, we address the inherent trade-offs between spatial and temporal precisions in the ultrafast nanoscopy. This method provides rich quantities of physical parameters, such as recombination time, photo-excited carrier density and EEA rates, yielding comprehensive understandings into carrier dynamics and excitonic behaviors through the nanometer and femtosecond scales. Our sideband-coupled GLIA method significantly enhances the signal-to-noise ratio by incorporating all harmonic components of the time-resolved near-field signals. It also overcomes the limitations of conventional homodyne detection, which typically requires extensive reference mirror scanning. This advancement promotes faster, more stable, and highly precise data acquisition, enabling the creation of a hyper-temporal mapping system that resolves time-resolved curves for every pixel with nm-fs spatiotemporal resolutions.

By utilizing this method, we characterize the nanoscale strain-induced spatiotemporal inhomogeneities such as the nano-bubbles that suppress the probability of bimolecular interaction of excitons by inducing band-distortion. Moreover, we visualized the topographically hidden defects such as the lattice mismatches that induce mid-gap states leading to trap photo-excited carriers and thereby to reduce the carrier densities and EEA rates, which in turn slows down recombination process. Our work bridges the gap between far-field pump-probe spectroscopy and nanoscale visualization by highlighting the potential of hyper-temporal nanoscopy to unravel the complex dynamics of material inhomogeneities in real space and time, offering new insights into semiconductor and optoelectronic applications and paving the way for future innovations in material design and characterization.

## 4 Materials and methods

### 4.1 Nanoscopy measurement

To obtain s-SNOM signals, we use a commercial photo-induced force microscope (PiFM)-based s-SNOM system (Vista One, Molecular Vista Inc.) equipped with a Michelson interferometer. The probe pulse is generated by a Ti:Sapphire oscillator (Mai Tai, Spectra-Physics, 780–820 nm, ∼80 MHz, ∼120 fs), which is then split by a beam splitter. Approximately 2 W of the probe pulse excites the OPO (MIRA OPO-X, Coherent Inc.) to generate the pump pulse. The pump pulse is modulated at *f*_pump_ (100 kHz) by an AOM which is controlled by the PiFM controller. The probe pulse path passes through a home-built time delay stage, the position of which is synchronized with the PiFM controllers. For the hyper-temporal imaging, we used pump and pulse fluence of approximately 0.6 mJ/cm^2^ and 0.03 mJ/cm^2^ corresponding to 6.25 pJ/pulse and 0.5 pJ/pulse, respectively. These values are based on estimated beam areas as ∼1.05 μm^2^ and 1.73 μm^2^, which are significantly smaller than the field of view (∼168.75 μm^2^), and the effective exposure time per pixel was approximately under 30 s. During the scanning, we did not observe any noticeable degradations of sample.

The attenuated pump and probe pulses illuminate the Pt–Ir coated tip (PPP-NCSTPt, Nanosensors), which vibrates with a 40 nm amplitude at *f*_tip_ (∼160 kHz) in PiFM tapping mode. The scattered tip signal and reference beam are collected by an APD (APD110A, Thorlabs) and filtered using a long-pass filter to block the high power of the pump beam. The interference signals ( $E_{\text{tr}}^{N}$ ⋅ *E* ^*R*^) are demodulated at ∼260 kHz, corresponding to the first sidebands (*f*_pump_) of the first harmonics (*f*_tip_), using the lock-in amplifier built into the PiFM controller. The reference mirror vibrates at *f*_M_ (∼277 Hz). The hyper-temporal scanning process is controlled using Python software.

### 4.2 Sample preparation

High-purity (>99.995 %) MoSe_2_ crystals were purchased from HQ graphene, with no additional dopants introduced during the growth process. Prior to exfoliation, silicon wafers with a 300-nm thick silicon oxide layer, used as substrates, were cleaned in an ultrasonication bath with acetone, isopropanol and subsequent immersion in piranha solution (H_2_SO_4_:H_2_O_2_ = 3:1) to remove organic residues. Multi-layered MoSe_2_ was mechanically exfoliated from bulk MoSe_2_ crystals and transferred onto the silicon oxide substrate.

## Associated content

**Supporting Information**. Supplementary Notes 1–4, Figures S1–S8, Extended Data 1,** **References.

Interferometric depression of far-field and time-independent near-field signals, details of GLIA method, the finite dipole modeling, method for EEA fitting, mapping of hotspots from pump and probe beams, simulation for dielectric function,** **a function of free electrons,** **time-resolved phase spectrum, and time-resolved s-SNOM images are explained. The Extended Data 1 is a video of time-resolved s-SNOM signals (file type: mp4).

## Supplementary Material

Supplementary Material Details
