# Supplementary material for: Characterizing nanoscale spatiotemporal defects of multi-layered MoSe2 in hyper-temporal transient nanoscopy
Source: Nanophotonics. 2025 Jun 24;14(15):2683–93. doi: 10.1515/nanoph-2025-0163 (PMC12322730; doi:10.1515/nanoph-2025-0163)
Supplement: Supplementary file 1 — Supplementary Material Details [file j_nanoph-2025-0163_suppl_001.pdf]

Supporting Information for

Characterizing Nanoscale Spatiotemporal Defects of Multi-layered $MoSe_2$ in Hyper-temporal Transient Nanoscopy

Hwi Je Woo[1†], Sung-Gyu Lee[2†], Hansung Kim[1], Suyong Jung[1], Eun Seong Lee[1] and Junghoon Jahng[1*]*

[1]Material Property Metrology Group, Korea Research Institute of Standards and Science (KRISS), Daejeon 34113, Republic of Korea

[2]School of Electrical and Electronic Engineering, Nanyang Technological University, Singapore, 639798, Singapore.

Keywords: transient s-SNOM, sideband-coupled GLIA, hyper-temporal, exciton, EEA

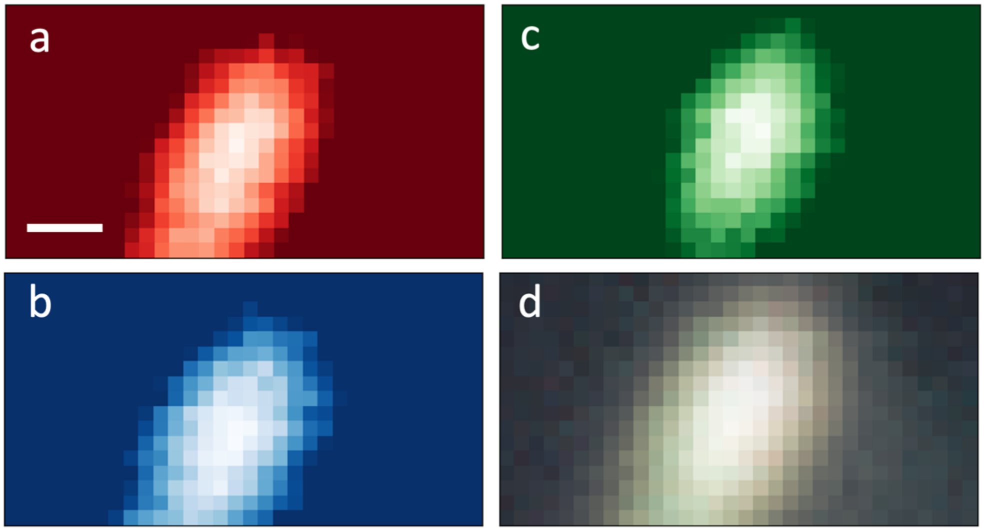

Figure S1. Validation for near-field hotspot of the pump and probe beam with photo-induced force mapping. We scan around the tip-apex by X-Y axis sweeping the parabolic mirror to get hotspots where the near-field is confined and enhanced by incident light. **(a)** and **(b)** Hotspot images of the probe pulse where the delay stage is fixed at -156.3 ps and 140.3 ps, respectively. The scale bar is 1 μm. **(c)** Hotspot images of the pump pulse. **(d)** Sum colored map of (a), (b) and (c). It shows that hotspots of the pump and probe pulses are spatially well overlapped.

Supporting Note 1: Interferometric depression of far-field signals and separation of time dependent/independent near-field signals in pump-probe s-SNOM with sideband-coupled GLIA method.

As mentioned in the main text, the sideband-coupled GLIA method effectively separates $E_{tr}^{N}(\Delta t)$ from the time-independent background signals including far-fields. The total intensity I^{total} obtained from the APD can be described as[1,2]:

$$\boldsymbol{I^{total}} = |\boldsymbol{E^N} + \boldsymbol{E^F} + \boldsymbol{E^R}|^2, \qquad (\mathbf{S1})$$

where $E^N = E_{tr}^{N}(\Delta t) + E_{probe}^{N} + E_{pump}^{N}$ and $E^F = E_{tr}^{F}(\Delta t) + E_{probe}^{F} + E_{pump}^{F}$. E_{probe}^{N} and E_{pump}^{N} represent the time-independent near-fields generated by the probe and pump, respectively, just as E_{probe}^{F} and E_{pump}^{F} represent the time-independent far-field. For simple expression, we assumed $m = 1$. In heterodyne detection[1], we are primarily interested in the product of E^N and E^R, since only the term $E^N \cdot E^R$ has the terms simultaneously amplitude modulated by f_{tip}, f_{pump}, and phase modulated by f_{M}. The term $E^F \cdot \bar{E}^R + E^R \cdot \bar{E}^F$ expands as:

$$2|E_{tr}^{F}||E^R|\cos(f_{pump}T)\cos(\phi_{tr}^{F} - \phi^R) + 2|E_{probe}^{F}||E^R|\cos(\phi_{probe}^{F} - \phi^R)$$
$$+2|E_{pump}^{F}||E^R|\cos(f_{pump}T)\cos(\phi_{pump}^{F} - \phi^R) \qquad (\mathrm{S2})$$

where $\bar{E}$ is conjugated complex value of E, and T is a time significantly larger than the delay time Δt. The last term is blocked by the LPF since it contains pump fields. The first term is eliminated as they are not modulated at f_{tip}, although they are modulated at f_{pump}. Thus, in case of $f_{\mathrm{DM}} = n \cdot f_{\mathrm{tip}} = f_{\mathrm{pump}}$, the first term is not clearly depressed[3–5]. Similarly, the term $E^N \cdot \bar{E}^F + E^F \cdot \bar{E}^N$ expands as:

$$2|E_{tr}^{N}||E_{tr}^{F}|\cos((nf_{tip} \pm f_{pump} - f_{pump})T)\cos(\phi_{tr}^{N} - \phi_{tr}^{F})$$
$$+2|E_{tr}^{N}||E_{probe}^{F}|\cos((nf_{tip} \pm f_{pump})T)\cos(\phi_{tr}^{N} - \phi_{probe}^{F})$$
$$+2|E_{probe}^{N}||E_{tr}^{F}|\cos((nf_{tip} - f_{pump})T)\cos(\phi_{probe}^{N} - \phi_{tr}^{F})$$
$$+2|E_{probe}^{N}||E_{probe}^{F}|\cos(nf_{tip}T)\cos(\phi_{probe}^{N} - \phi_{probe}^{F})$$
$$+2|E_{tr}^{N}||E_{pump}^{F}|\cos((nf_{tip} \pm f_{pump} - f_{pump})T)\cos(\phi_{tr}^{N} - \phi_{pump}^{F})$$

$$+2\left|E^{N}_{probe}\right|\left|E^{F}_{pump}\right|\cos\left((nf_{tip}-f_{pump})T\right)\cos\left(\phi^{N}_{probe}-\phi^{F}_{pump}\right)$$
$$+2\left|E^{N}_{pump}\right|\left|E^{F}_{tr}\right|\cos\left((nf_{tip}\pm f_{pump}-f_{pump})T\right)\cos\left(\phi^{N}_{pump}-\phi^{F}_{tr}\right)$$
$$+2\left|E^{N}_{pump}\right|\left|E^{F}_{probe}\right|\cos\left((nf_{tip}\pm f_{pump})T\right)\cos\left(\phi^{N}_{pump}-\phi^{F}_{probe}\right)$$
$$+2\left|E^{N}_{pump}\right|\left|E^{F}_{pump}\right|\cos\left((nf_{tip}\pm f_{pump}-f_{pump})T\right)\cos\left(\phi^{N}_{pump}-\phi^{F}_{pump}\right) \quad \text{(S3)}$$

The 5th–9th terms are eliminated by the LPF, and the 4th term is not modulated by f_{pump}. In homodyne detection, the 1st–3rd terms can be neglected when E^{R} is much larger than E^{N} and E^{R}. However, the 1st–3rd terms are not perfectly especially in case of E^{F}_{total} is large. In contrast, by applying the pseudo-heterodyne or GLIA method, these terms can be entirely neglected because the phase modulation method eliminates far-field signals[2]. Thus, I^{total} can be simplified as $E^{N}\cdot\bar{E}^{R}+E^{R}\cdot\bar{E}^{N}$. Now, I^{total} can be expressed as:

$$I^{total}=2\left|E^{N}_{tr}\right|\left|E^{R}\right|\cos\left(\left(nf_{\text{tip}}+f_{\text{pump}}\right)T\right)\cos\left(\phi^{N}_{tr}-\phi^{R}\right)$$
$$+\,2\left|E^{N}_{probe}\right|\left|E^{R}\right|\cos\left(nf_{\text{tip}}T\right)\cos\left(\phi^{N}_{probe}-\phi^{R}\right)$$
$$+2\left|E^{N}_{pump}\right|\left|E^{R}\right|\cos\left((nf_{tip}\pm f_{pump}T\right)\cos\left(\phi^{N}_{pump}-\phi^{R}\right) \quad \text{(S4)}$$

The last term is eliminated by LPF, and the second term is not modulated by the pump pulse. Note that, in the case of non-sideband coupled system ($f_{\text{pump}}=0$ or $n\cdot f_{\text{tip}}$, and $f_{\text{DM}}=n\cdot f_{\text{tip}}$), the second term is not clearly depressed[3–5]. Since the first term is modulated by f_{tip}, and f_{pump}, the time dependent near-field $I^{N}_{tr}(\Delta t)$ can be written as:

$$I^{N}_{tr}(\Delta t)=2\left|E^{N}_{tr}\right|\left|E^{R}\right|\cos(\Omega T)\cos\left(\phi^{N}_{tr}-\phi^{R}\right) \quad \text{(S5)}$$

where Ω is $nf_{\text{tip}}\pm f_{\text{pump}}$. In the case of pseudo-heterodyne, the demodulated amplitudes of sidebands are expressed:

$$X_{I(\Delta t)}=\frac{1}{f_{M}T_{int}}\int_{0}^{f_{M}T_{int}}I^{N}_{tr}(\Delta t)\cos(f_{M}T)\,d(f_{M}\mathrm{T}),$$
$$Y_{I(\Delta t)}=\frac{1}{f_{M}T_{int}}\int_{0}^{f_{M}T_{int}}I^{N}_{tr}(\Delta t)\sin(f_{M}T)\,d(f_{M}\mathrm{T}), \quad \text{(S6)}$$

and using Jacobi-Anger expansion:

$$X_{I(\Delta t)}=\;E_{R}E^{N}_{tr}\cos\phi^{N}_{tr}\left(J_{l}(a)\right)\,for\ l\ is\ even$$
$$Y_{I(\Delta t)}=E_{R}E^{N}_{tr}\sin\phi^{N}_{tr}\left(J_{l}(a)\right)\,for\ l\ is\ odd \quad \text{(S7)}$$

Where J_n is n^{th} Bessel function, a is phase modulation amplitude and l is the harmonic order of sidebands of Ω. Thus, pseudo-heterodyne method picks up only the first (odd) and second (even) sidebands of Ω signals to retrieve near-field amplitude and phase signals by implementing two lock-ins. On the other hands, the GLIA method integrates over the sidebands of Ω signals by multiplying the two orthogonal reference signals $C(T) = 2\cos(\Omega T)\cos\phi^R$ and $S(T) = 2\cos(\Omega T)\sin\phi^R$ to $I_{tr}^N(\Delta t)$, we obtain[6]:

$$X_{I(\Delta t)} = \frac{1}{f_M T_{int}} \int_0^{f_M T_{int}} I_{tr}^N(\Delta t)\, C(T) d(f_M \mathrm{T}),$$

$$Y_{I(\Delta t)} = \frac{1}{f_M T_{int}} \int_0^{f_M T_{int}} I_{tr}^N(\Delta t)\, S(T) d(f_M \mathrm{T}) \qquad \text{(S8)}$$

where T_{int} is the integration time of signals. The meaning of Eq. (S8). is the sum of orthogonal components across all sideband frequencies, enabling comprehensive signal extraction using the GLIA method. Consequently, by setting $\phi^R = a\sin f_\mathrm{M} T$, where a is modulation depth of the phase, we clearly retrieve the two orthogonal signals as[6]:

$$X_{I(\Delta t)} = E_R E_{tr}^N \cos\phi_{tr}^N \left(1 + J_{2n}(2a) + J_0(2a)\right),$$

$$Y_{I(\Delta t)} = E_R E_{tr}^N \sin\phi_{tr}^N \left(1 - J_{2n}(2a) - J_0(2a)\right) \qquad \text{(S9)}$$

In our case, we choose $a \approx 2.404$ rad where $J_0(a) = 0$ for simple calculation of time-resolved amplitude and phase. Finally, $I(\Delta t)$ and $\phi(\Delta t)$ are extracted from the Eq. S7. Unlike pseudo-heterodyne detection, GLIA employs reference signals encompassing all harmonic frequencies, allowing for maximizing signal to noise ratio.

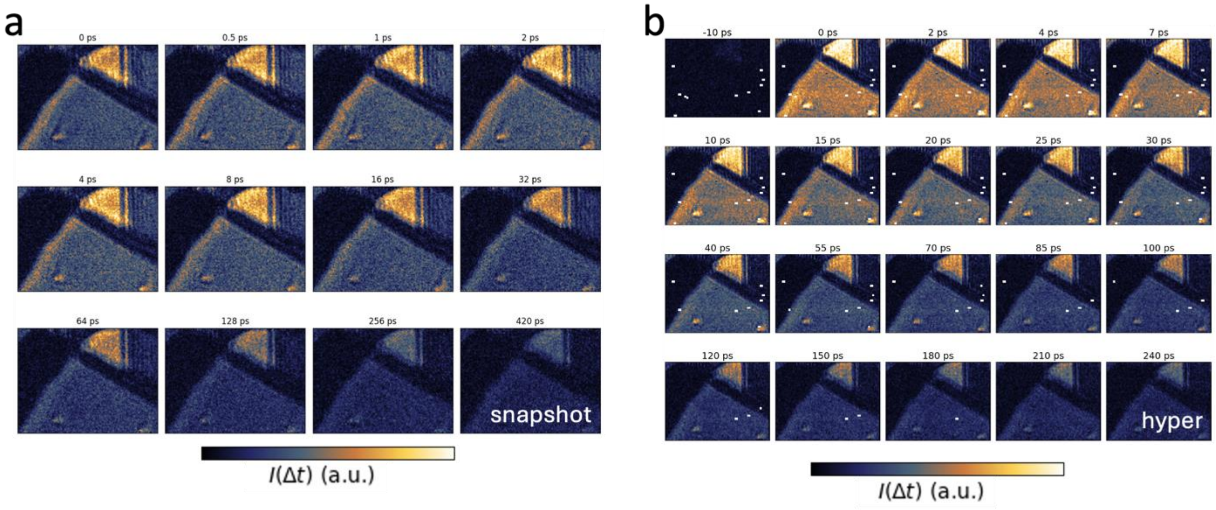

Figure S2. Time-resolved s-SNOM images. (a) Obtained using the snapshots method. **(b)** Extracted from the hyper-temporal data in Extended data 1.

Supporting Note 2: Separation of E_{tr}^{N} from E_{tr}^{F} and E_{probe}^{N} in sideband-coupled GLIA method.

Figures S3a illustrates the far-field interference effect commonly encountered in non-uniform samples. In the presence of large reflectors or scatterers such as large wrinkles or bumps, the time-resolved far-field E_{tr}^{F} (blue) contribution from the combination between the pump and probe beams cannot be neglected. This issue can be overcome by setting $f_{\text{pump}} \neq n \cdot f_{\text{tip}}$ and $f_{\text{pump}} \neq l \cdot f_{\text{M}}$, which effectively separates E_{tr}^{F} from E_{tr}^{N} because the sideband-coupled signal at the frequency $f_{\text{tip}} \pm f_{\text{pump}}$ is not affected by E_{probe}^{N} as shown in Figure S3c. Furthermore, the interaction of the tip-enhanced pump and probe beams near the tip produces near-field signals at the mixed frequencies $f_{\text{tip}} \pm f_{\text{pump}}$ with E^{F} excluded[7]. To prove the suppression and decomposition of E_{tr}^{F} and E_{probe}^{N} using the sideband-coupled GLIA, time-resolved point spectroscopy was conducted at the marked position 'a' and 'b' in the inset image of Figure S3a. These positions locate near a vertical wrinkle line of approximately 100 nm high, acting as a strong scatterer. While the method of references [[3–5]], shows a deep dip in Figure S3d at both positions resulting from the interference between E_{probe}^{N} and E_{tr}^{F}, Figure S3e from sideband-coupled GLIA clearly reveals exponential decay, indicating successful separation of E_{tr}^{N} from E_{tr}^{F} and E_{probe}^{N}.

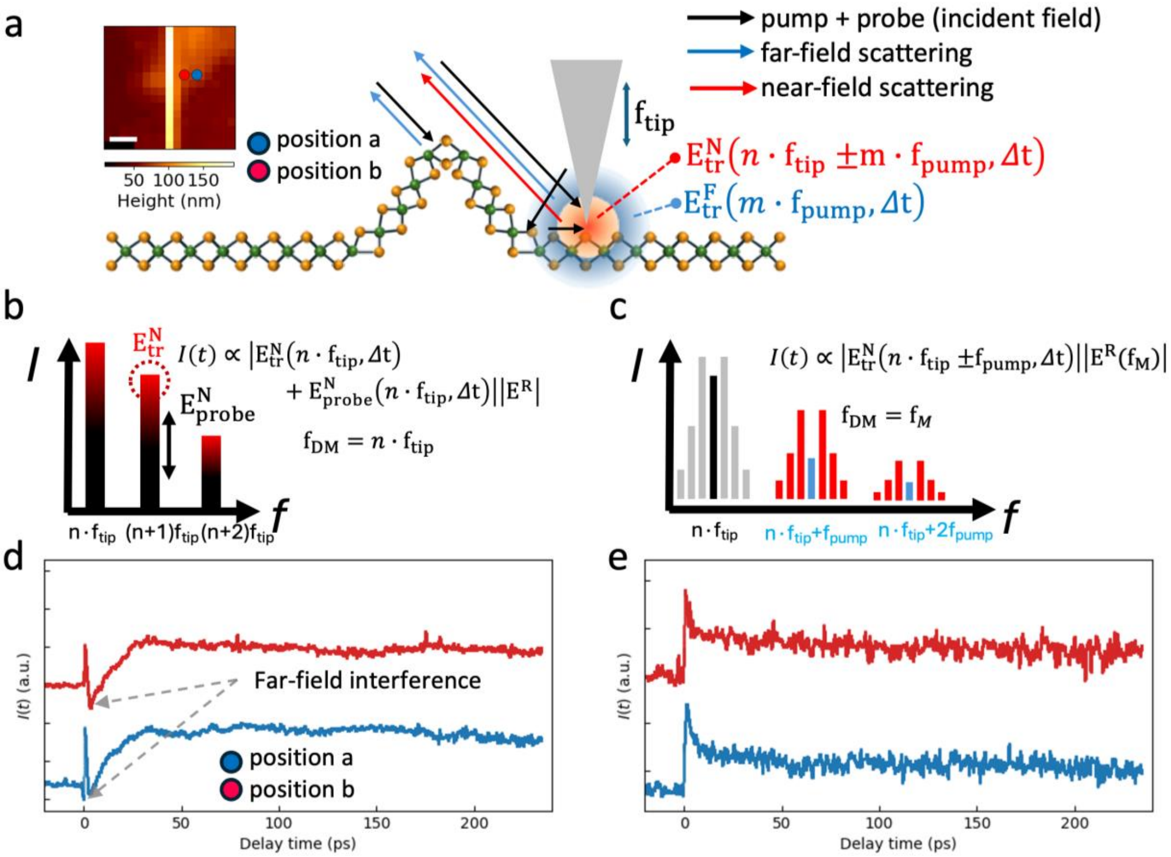

Figure S3. Validation of frequency separation between far-fields and ground-state/photo-excited near-fields in the sideband-coupled GLIA technique. (a) Illustration of photo-excited far- and near-fields around the tip and the large scatterer (inset: AFM topographic image of the large wrinkle in $MoSe_2$, scalebar: 500 nm). **(b)** Frequency spectrum under conditions where pump is modulated at n^{th} harmonics of f_{tip}, with the signals demodulated at n^{th} harmonics of f_{tip} (f_{DM}: demodulation frequency, $f_{DM} = n \cdot f_{tip} = f_{pump}$). **(c)** Frequency spectrum under conditions of the sideband-coupled GLIA technique. **(d)** Time-resolved spectra at the positions 'a' and 'b' under the condition of $f_{DM} = n \cdot f_{tip} = f_{pump}$. **(e)** Time-resolved spectra under the sideband-coupled GLIA technique. Spectra in (d) and (e) are vertically shifted for clarity. Time-resolved spectra obtained by pumping at 1.97 eV and probing at 1.55 eV.

Supporting Note 3: The finite dipole modeling in multi-layered $MoSe_2$.

As mentioned in the main text, to describe the tip-scattered near-field signal, we employ the finite dipole model[8] as shown in Fig S4. The tip is assumed as an ellipsoid with length of $2L$. The H is the tip-sample gap distance and d_l is the thickness of the $MoSe_2$. When the light illuminating (E_0) the tip, the initial charge Q_0 and potential induced charge Q_1 are positioned in the tip. We can approximate the potential response by point charges in cylindrical coordinate as:

$$U = -\frac{Q_0}{4\pi\varepsilon_0}\left(\Phi + \frac{1}{\sqrt{r^2 + z^2}}\right) \tag{S10}$$

where, $A(k) = \frac{\beta_{01}+\beta_{12}e^{-2kd_1}}{1-\beta_{10}\beta_{12}e^{-2kd_1}}e^{-2kz_0}$ and $\beta_{ij} = \frac{\varepsilon_i-\varepsilon_j}{\varepsilon_i+\varepsilon_j}$ (i = 0, 1, and 2). The image charge in the sample is given by $Q_0' = -\beta_X Q_0$ at the distance X_0 under the sample surface (boundary condition: $\beta_X = \frac{\Phi_1^2}{\Phi'_1}|_{z=0}$ and $X = -\frac{\Phi_1}{\Phi'_1}|_{z=0}$). By multiple-scattering process between Q_0', Q_1 and Q_0:

$$Q_1 = \beta_{X_0} f_0 Q_0 + \beta_{X_1} f_1 Q_1 \tag{S11}$$

where $Q_0 = 4\pi R^2 E_0$, $\beta_{X_{0,1}} = -\frac{\Phi_1(z_{0,1})^2}{\Phi'_1(z_{0,1})}|_{z=0}$, $f_{0,1} = \left(g - \frac{R+2H+z_{0,1}}{2L}\right)\frac{\ln\frac{4L}{R+4H+2z_{0,1}}}{\ln\frac{4L}{R}}$ and g is the empirical geometric factor (mostly ~0.7). Finally, we can get the effective polarizability α_{eff} as:

$$\alpha_{eff} \propto \frac{1}{2}\frac{\beta_{X_0} f_0}{1 - \beta_{X_{0,1}} f_1} + 1 \tag{S12}$$

In this study, the effective polarizability α_{eff} is described using parameters where the radius of the tip is 30 nm, demodulation tapping amplitude (A_{tip}) with 20 nm at 1st harmonics, and the angle of incident light is 60˚. The demodulation is account as:

$$H(t) = H + A_{tip}\left(1 + \cos f_{tip} T\right). \tag{S13}$$

As shown in the Fig. S4, for a multi-layered system, the far-field reflection coefficient $r_p(\Delta t)$ is calculated using the transfer matrix method and is described as following equation[9]:

$$r_p = \frac{r_{01} + r_{12}e^{2i\xi}}{1 + r_{01}r_{12}e^{2i\xi}}, \tag{S14}$$

$$k_i = \frac{2\pi}{\lambda}\sqrt{\varepsilon_i - \sin^2\theta}\,, i = 0, 1, and\ 2 \tag{S15}$$

$$\xi = k_1 d_1. \tag{S16}$$

Where the d_1 is the thickness of the sample and θ is the angle of incident light. Therefore, $I(\Delta t)$ depends not only on the effective polarizability but also on multi-reflection effects within the multi-layered sample, which are influenced by the sample thickness.

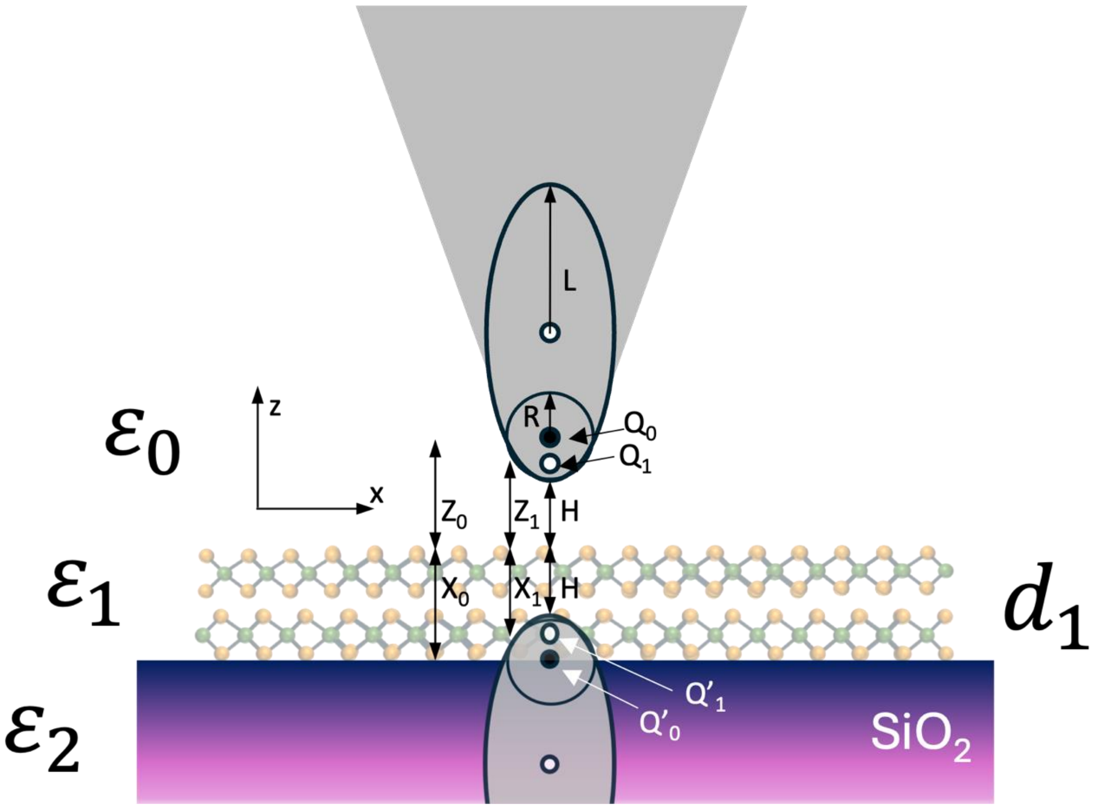

Figure S4. Geometry of the sample for the finite dipole model.

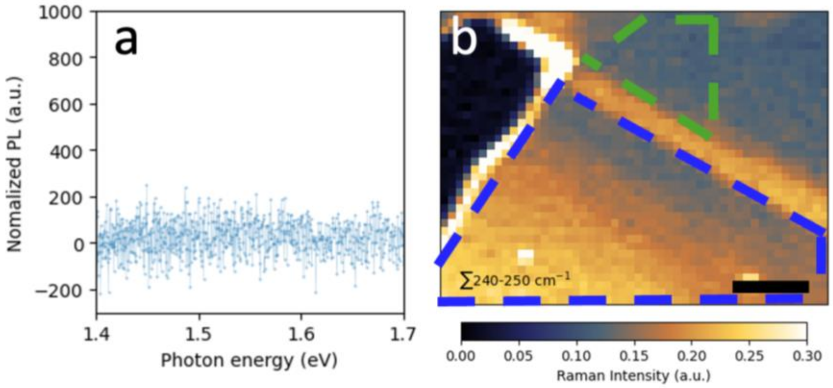

Figure S5. Far-field spectroscopy. (a) Photoluminescence result. **(b)** Raman spectroscopy mapping results, summing all intensities between 240–250 cm^{-1}, corresponding to the A_{1g} mode. The scalebar represents 3 μm.

Supporting Note 4: Linearized s-SNOM intensity and fitting for EEA.

To determine τ_{ex} and N_0^{2d}, we used the relation $dn_{ex}/dt = -\tau_{ex} \cdot N_0^{2d} \cdot n_{ex}$ in conjunction with Eqs. (2) and (3). To obtained EEA rates discussed in the main text, we linearized the s-SNOM intensity as described by the following equation[10]:

$$N(\Delta t) = \frac{N_0}{1 + k_{EEA} N_0 \Delta t} \tag{S17}$$

Where k_{EEA} is the EEA rates. To linearize s-SNOM signals, we assumed that the s-SNOM signal is proportional to the photo-excited carrier densities and that EEA dominates the early decay process within 0–25 ps time window. This assumption leads to the following equation:

$$\frac{N_0}{N_t} - 1 = k_{EEA} N_0 \Delta t \tag{S18}$$

From Equation S9, we fit all pixels in the hyper-temporal data to extracts k_{EEA} for each pixel.

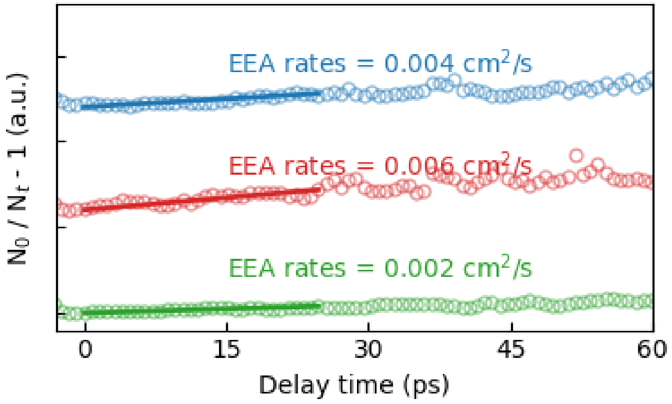

Figure S6. Linearized s-SNOM signals and fitting results using Eq. (S18).

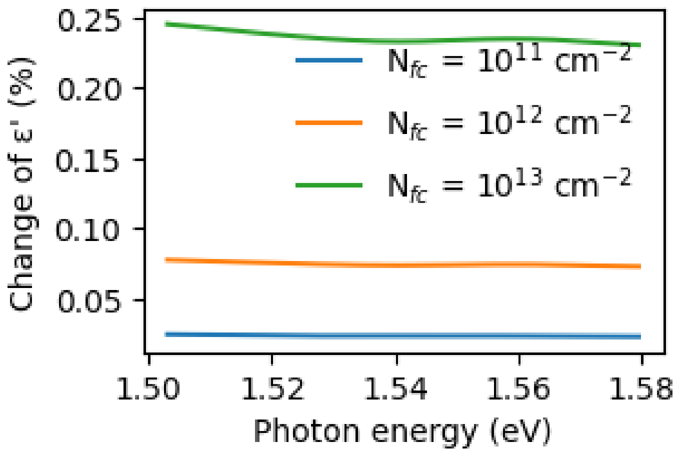

Figure S7. Change in the dielectric function as a function of free electron density in the Drude–Lorentz model.

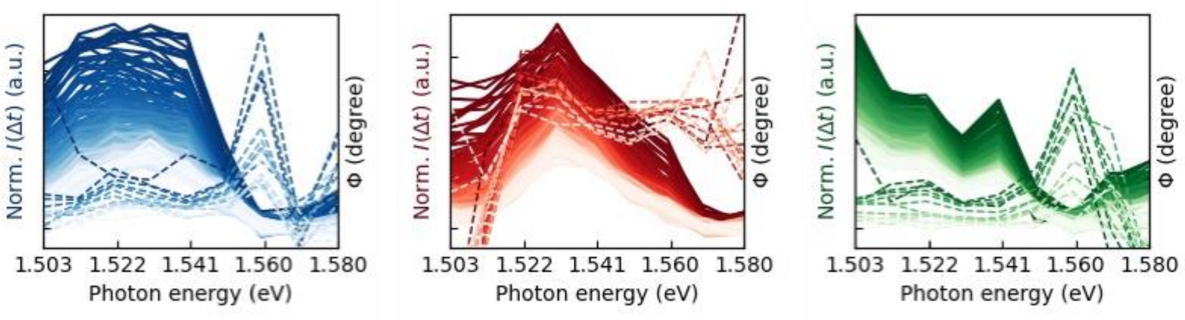

Figure S8. Time-resolved near-field phase (dashed) corresponded to Fig. 4g, 4h and 4i. The phase data are averaged section of 25 ps. Phase data show clear dissipative shapes, except strained position (red).

References

(1) Chen, X.; Hu, D.; Mescall, R.; You, G.; Basov, D. N.; Dai, Q.; Liu, M. Modern Scattering-Type Scanning Near-Field Optical Microscopy for Advanced Material Research. *Advanced Materials* **2019**, *31* (24), 1804774. https://doi.org/10.1002/adma.201804774.

(2) Ocelic, N.; Huber, A.; Hillenbrand, R. Pseudoheterodyne Detection for Background-Free near-Field Spectroscopy. *Appl. Phys. Lett.* **2006**, *89* (10). https://doi.org/10.1063/1.2348781.

(3) Li, J.; Yang, R.; Higashitarumizu, N.; Dai, S.; Wu, J.; Javey, A.; Grigoropoulos, C. P. Transient Nanoscopy of Exciton Dynamics in 2D Transition Metal Dichalcogenides. *Advanced Materials* **2024**, *36* (21), 2311568. https://doi.org/10.1002/adma.202311568.

(4) Li, J.; Yang, R.; Rho, Y.; Ci, P.; Eliceiri, M.; Park, H. K.; Wu, J.; Grigoropoulos, C. P. Ultrafast Optical Nanoscopy of Carrier Dynamics in Silicon Nanowires. *Nano Letters* **2023**. https://doi.org/10.1021/acs.nanolett.2c04790.

(5) Yang, R.; Li, J.; Cai, Y.; Blankenship, B. W.; Wu, J.; Grigoropoulos, C. P. Near-Field Nanoimaging of Phases and Carrier Dynamics in Vanadium Dioxide Nanobeams. *ACS Photonics* **2024**. https://doi.org/10.1021/acsphotonics.4c00848.

(6) Al Mohtar, A.; Vaillant, J.; Sedaghat, Z.; Kazan, M.; Joly, L.; Stoeffler, C.; Cousin, J.; Khoury, A.; Bruyant, A. Generalized Lock-in Detection for Interferometry: Application to Phase Sensitive Spectroscopy and near-Field Nanoscopy. *Opt. Express* **2014**, *22* (18), 22232. https://doi.org/10.1364/OE.22.022232.

(7) Plankl, M.; Faria Junior, P. E.; Mooshammer, F.; Siday, T.; Zizlsperger, M.; Sandner, F.; Schiegl, F.; Maier, S.; Huber, M. A.; Gmitra, M.; Fabian, J.; Boland, J. L.; Cocker, T. L.; Huber, R. Subcycle Contact-Free Nanoscopy of Ultrafast Interlayer Transport in Atomically Thin Heterostructures. *Nat. Photon.* **2021**, *15* (8), 594–600. https://doi.org/10.1038/s41566-021-00813-y.

(8) Hauer, B.; Engelhardt, A. P.; Taubner, T. Quasi-Analytical Model for Scattering Infrared near-Field Microscopy on Layered Systems. *Opt. Express, OE* **2012**, *20* (12), 13173–13188. https://doi.org/10.1364/OE.20.013173.

(9) Govyadinov, A. A.; Amenabar, I.; Huth, F.; Carney, P. S.; Hillenbrand, R. Quantitative Measurement of Local Infrared Absorption and Dielectric Function with Tip-Enhanced Near-Field Microscopy. *J. Phys. Chem. Lett.* **2013**, *4* (9), 1526–1531. https://doi.org/10.1021/jz400453r.

(10) Sun, D.; Rao, Y.; Reider, G. A.; Chen, G.; You, Y.; Brézin, L.; Harutyunyan, A. R.; Heinz, T. F. Observation of Rapid Exciton–Exciton Annihilation in Monolayer Molybdenum Disulfide. *Nano Lett.* **2014**, *14* (10), 5625–5629. https://doi.org/10.1021/nl5021975.
